# Editorial: Locus of Control: Antecedents, Consequences and Interventions Using Rotter's Definition

**DOI:** 10.3389/fpsyg.2021.698917

**Published:** 2021-06-29

**Authors:** Stephen Nowicki, Yasmin Iles-Caven, Ari Kalechstein, Jean Golding

**Affiliations:** ^1^Department of Psychology, Emory University, Atlanta, GA, United States; ^2^Bristol Medical School, Public Health Sciences, University of Bristol, Bristol, United Kingdom; ^3^Executive Mental Health, Inc., Los Angeles, CA, United States

**Keywords:** locus of control, Rotter's concept of locus of control, ALSPAC, antecedents of locus of control, longitudinal cohort, parental effects of locus of control, child outcomes

Locus of control (LOC) is at the same time, one of the most popular and yet one of the most misused personality attributes in the social sciences. It was introduced into psychology in 1966 by Julian Rotter who conceptualized it as a generalized expectancy within his Social Learning Theory and defined it as follows:

“Internal vs. external control refers to the degree to which persons expect that a reinforcement or an outcome of their behavior is contingent on their own behavior or personal characteristic vs. the degree to which persons expect that the reinforcement or outcome is a function of chance, luck, or fate, is under the control of powerful others or is simply unpredictable. Such expectancies may generalize along a gradient based on the degree of semantic similarity of the situational cues.” (Rotter, 1966).

Although the number of studies with LOC as a major variable reaches into the thousands and research continues at a brisk pace up to the present day across disciplines, the way in which investigators have eroded, ignored, and misapplied Rotter's original definition of LOC is cause for scientific concern. Without an agreed upon definition of LOC and reliable ways of measuring it based on that definition, generalization across studies becomes difficult if not impossible.

The purpose of studies completed within this topic was to use Rotter's definition of LOC and measures of LOC consistent with that definition to investigate (1) the stability and change of children's and adults' LOC over time; Nowicki et al.(a); (2) antecedents of children's and adults' LOC [Carton et al.; Nowicki et al.(b)]; (3) the association of parents' prenatal LOC with children's academic and social outcomes [Golding, Gregory, Ellis, Nunes, et al.; Nowicki et al.(c); Golding, Gregory, Ellis, Iles-Caven, et al.]; (4) the association between change in parents LOC over time (6 years) and children's social success or failure [Nowicki et al.(d); Nowicki et al.(e)]; (5) the associations of children's LOC and internalizing and externalizing problems (Flores et al.); depression (Costantini et al.; Sullivan et al.) and epilepsy (Wolf et al.); and (6) the viability of interventions focused on changing LOC (Tyler et al.).

Researchers within this topic gathered data by using construct valid tests for adults and children developed to be consistent with Rotter's definition of LOC as a generalized expectancy. Although some past studies have used measures of LOC that were dubious (e.g., one or two items plucked from a non-LOC scale) to evaluate the validity of LOC, the present studies were among the first to produce longitudinal information about the stability over time of LOC in children and adults (more stable in adults than children) and the impact of prenatal parental LOC on children's subsequent outcomes. Researchers also found that the greater the degree of externality in prenatal parents' LOC, the more negative were the children's outcomes in sleeping, eating, and emotional lability early in life and social/emotional adjustment and cognitive performance later in childhood. The association of prenatal parental LOC with children's outcomes was further supported by findings showing a significant association between parents' change toward internality over time (6 years) and more positive social and academic outcomes in their children when compared to parent child outcomes associated with parent LOC that remained the same or became more external over time.

Findings that parents' LOC is associated with children's outcomes suggests looking at the possibility that interventions focused on changing parental externality before children are born may be worthwhile. Support for this possibility was found in results indicating parental change toward internality was associated with positive child outcomes (as reflected in children's personal and social outcomes as rated by teachers). Results from another study indicated improvements in the parental relationship and improvement in their economic conditions were associated with parents becoming more internal. Although, cause and effect cannot be assigned, the findings suggest future research should be directed at evaluating if strengthening the parental dyad relationship and improving the family financial situations would result in parents changing toward internality and children's outcomes becoming more positive.

Other topic studies revealed more about possible parental behaviors, attitudes and actions related to children's LOC. Since, the last review of parental antecedents of children's LOC was published over a quarter century ago, a recent update was needed. What it found was that parents disciplinary actions characterized by authoritative approaches and parents more often contingently reinforcing their children's behavior/outcome sequences (as observed in laboratory interactions) are associated with greater internality. However, since there have been only a few observational studies of parent child interactions there is a need for more investigations spanning children at different ages of development.

A final set of studies within the LOC topic gathered information on associations between children's LOC and their personal, social, and physical outcomes. A longitudinal study of Spanish speaking children in northern Chile produced similar associations between children's externality and a greater frequency of internalizing and externalizing problems to those found previously with English speaking participants. Other studies revealed how internal LOC acts as a mediator to buffer against the development of depression in young high-risk children from compromised environments; a result found in high-risk adolescent children as well. Considering the LOC, depression association, the topic study that focused on a strength-based intervention with offenders to improve their LOC may have relevance for other populations of children. In any case, there is a general need for research to illuminate LOC antecedents as possible targets for inclusion in intervention programs to help children develop internality as a way to prevent depression.

A final study dealt with the impact of a chronic disease, in this case epilepsy, on children's LOC. When children experience a serious disease and/or disability like epilepsy they may erroneously “learn” to be more helpless than they actually are to deal with the affliction and its consequences. Children need the help of caretakers to learn the full impact of what outcomes their behavior is tied to so they can be active participants not only in their treatment, but in shaping their lives outside of treatment.

The take home message from this set of studies is that LOC as defined by Rotter and measured with scales consistent with his definition remains an important construct. The degree to which individuals view the connection between what they do and what happens to them appears to have relevance for parents expecting a child and in children dealing with social interactions, academic achievement, and/or chronic mental or physical disease. Because of the findings, more research closely tied to Rotter's social learning theory is needed to identify relevant antecedents of LOC expectancies and valid interventions to help children and adults learn to develop the full extent of their internality.

## Author Contributions

All authors listed have made a substantial, direct and intellectual contribution to the work, and approved it for publication.

## Conflict of Interest

The authors declare that the research was conducted in the absence of any commercial or financial relationships that could be construed as a potential conflict of interest.
